# Nodal Na^+^ and Ca^2+^ flux dynamics in cortical myelinated axons

**DOI:** 10.3389/fncel.2025.1662730

**Published:** 2025-09-03

**Authors:** Oron Kotler, Kenichi Miyazaki, Yana Khrapunsky, William N. Ross, Ilya A. Fleidervish

**Affiliations:** 1 Department of Physiology and Cell Biology, Faculty of Health Sciences and Zelman Center for Brain Science Research, Ben–Gurion University of the Negev, Beer Sheva, Israel; 2 Department of Physiology, New York Medical College, Valhalla, NY, United States

**Keywords:** neocortex, pyramidal neuron, myelinated axon, node of Ranvier, Na^+^ channel, Ca^2+^ channel, fluorescence imaging

## Abstract

Functional neuronal connectivity relies on long-range propagation of action potentials by myelinated axons. This process critically depends on the distribution and biophysical properties of ion channels clustered at specialized, regularly spaced domains, the nodes of Ranvier, where the signals are actively regenerated. Morphological and functional evidence indicates that voltage-gated Na^+^ channels, which directly support action potential conduction, are exclusively localized at nodes. While these domains also contain voltage-gated Ca^2+^ channels that contribute to key intracellular signaling cascades, evidence regarding the presence of functional Ca^2+^ channels in the internodal regions remains conflicting. Using high-speed fluorescence imaging, we characterized action potential–evoked Na^+^ and Ca^2+^ dynamics at the nodes of Ranvier in myelinated axons of layer 5 pyramidal neurons in cortical brain slices. Spatially, both Na^+^ and Ca^2+^ elevations were largely restricted to the nodal regions. The time-to-peak of the nodal Na^+^ transients was significantly shorter (3.7 ± 0.3 ms) than that of the Ca^2+^ transients (10.3 ± 0.6 ms with OGB-1, 4.2 ± 0.5 ms with OGB-5 N), consistent with electrophysiological evidence indicating that Na^+^ influx occurs primarily during the action potential upstroke, whereas Ca^2+^ influx predominantly takes place during and after the repolarization phase. The decay of Na^+^ transients, reflecting lateral diffusion into the internodes, was exceptionally fast in short nodes and became progressively slower in longer ones, consistent with computational models assuming diffusion-based clearance alone. In contrast, Ca^2+^ transients decayed more slowly and showed no dependence on nodal length, consistent with clearance dominated by active transport. Finally, the post-spike recovery of nodal Na^+^ fluxes was rapid and temperature-dependent, consistent with the reactivation kinetics of voltage-gated Na^+^ channels. In contrast, the similarly rapid but temperature-independent recovery of Ca^2+^ flux suggests that a single action potential does not induce Ca^2+^ channel inactivation and therefore has minimal impact on their availability during subsequent spikes.

## 1 Introduction

The nodes of Ranvier, specialized domains within myelinated axons essential for action potential propagation, are known to contain both voltage-gated Na^+^ (Fleidervish et al., 2010; Rasband and Peles, 2021) and Ca^2+^ (Grundemann and Clark, 2015; Zhang and David, 2016; Hanemaaijer et al., 2020) channels. In CNS axons, Na^+^ channels directly support action potential conduction (Hille, 1984), while Ca^2+^ channels are thought to mediate intracellular signaling cascades, affecting Ca^2^-sensitive K^+^ channels, mitochondria, and axo-glial communications (Augustine et al., 2003). However, due to the technical challenges of performing electrophysiological and imaging recordings in ultra-thin central axons, the precise distribution and kinetics of these channels remain elusive.

Nav1.6 channels are known to localize at nodes of Ranvier, and are not found in the internodal membrane (Caldwell et al., 2000; Peles and Salzer, 2000). Nodal localization of Na^+^ channels is due to their preferential anchoring by the nodal ankyrin G complexes (Rasband and Peles, 2015, 2021), and is confirmed by both immunohistochemistry (Boiko et al., 2001) and by high-speed fluorescence imaging (Fleidervish et al., 2010). In contrast, the precise molecular mechanisms responsible for anchoring Ca^2+^ channels remain unknown, and evidence is conflicting regarding the presence of functional Ca^2+^ channels in the internodal regions (Grundemann and Clark, 2015; Zhang and David, 2016; Hanemaaijer et al., 2020).

Functionally, Na^+^ channels exhibit rapid activation and inactivation kinetics, resulting in a steep rise in open probability during the action potential upstroke and abrupt current termination near the spike peak (Hille, 1984). In hippocampal mossy fibers and proximal axons of cortical pyramidal neurons, this minimizes overlap between Na^+^ current and the activation of slower voltage-gated K^+^ channels (Alle et al., 2009; Hallermann et al., 2012), thereby enhancing the energy efficiency of action potential signaling. However, it remains unclear whether the kinetics of nodal Na^+^ channels and the shape of the nodal action potential resembles those observed in other neuronal compartments. In presynaptic terminals, Ca^2+^ channels exhibit markedly slower activation and inactivation kinetics than Na^+^ channels, resulting in a substantial portion of Ca^2+^ influx occurring via prominent tail currents (Augustine et al., 2003). Therefore, calcium entry persists even after the membrane potential has returned to rest. It remains unclear whether the open probability of nodal Ca^2+^ channels follows a similar pattern, and whether individual action potentials induce inactivation of these channels.

Here, using high speed imaging of fluorescence changes of Na^+^ and Ca^2+^ indicators, we characterized Na^+^ and Ca^2+^ fluxes at cortical nodes of Ranvier. We found that the spatial extent of these fluxes was variable and that there was a complex interplay between their distinct channel dynamics. While nodal Na^+^ channels undergo rapid inactivation and recovery following an action potential, nodal Ca^2+^ channels do not inactivate. Consequently, a transient reduction in Ca^2+^ influx reflects a decrease in action potential amplitude, arising from incomplete recovery of Na^+^ channel availability.

## 2 Materials and methods

Experiments were conducted at Ben-Gurion University of the Negev and the Marine Biological Laboratory in Woods Hole, MA, in accordance with established guidelines for the welfare of experimental animals. All procedures were approved by the Institutional Animal Care and Use Committees of Ben-Gurion University and the Marine Biological Laboratory.

Experiments were performed on L5 pyramidal neurons in 300-μm-thick neocortical slices prepared from the P23-P60 mouse brain as previously described (Fleidervish et al., 2010). Mice of either sex were anesthetized with isoflurane and then decapitated. The brains were placed in cold (4–8 °C) oxygenated (95% O_2_–5% CO_2_) artificial cerebrospinal fluid (aCSF). Slices (300 μm) were cut on a vibratome (VT1200, Leica) and placed in a holding chamber containing oxygenated aCSF at room temperature; they were transferred to a recording chamber after at least 30 min of incubation. The composition of the aCSF was (in mM): 124 NaCl, 3 KCl, 2 CaCl_2_, 2 MgSO_4_, 1.25 NaH_2_PO_4_, 26 NaHCO_3_, and 10 glucose (all chemicals obtained from Sigma Aldrich); pH was 7.4 when bubbled with 95% O_2_/CO_2_.

### 2.1 Electrophysiology

Cells were viewed with a 60 × water-immersion lens in a BX51WI microscope (Olympus) mounted on an X–Y translation stage. Somatic whole-cell recordings were made using patch pipettes pulled from thick-walled borosilicate glass capillaries (1.5-mm outer diameter; Sutter Instruments, CA). Pipettes had a resistance of 5 to 9 MΩ when filled with K-gluconate based solution with the following composition (in mM): K gluconate 134, KCl 6, NaCl 4, MgCl_2_ 2, HEPES 10 (pH 7.25 at 22 °C). In all experiments, the solution was supplemented with one of two Na^+^-sensitive dyes: sodium-binding benzofuran isophthalate (SBFI, 2 mM; Molecular Probes, OR), or ANG-2 (0.4 mM; TEFLabs, TX), along with one of the following Ca^2+^-sensitive dyes: Oregon Green BAPTA-1 (OGB-1, 100 μM; Thermo Fisher Scientific, MA), Oregon Green BAPTA-5 N (OGB-5 N, 100 μM; Thermo Fisher Scientific, MA), or bis-fura-2 (300 μM; Thermo Fisher Scientific, MA). Recordings were made using a MultiClamp 700B amplifier equipped with a hybrid CV-7B headstage (Molecular Devices, CA) in current clamp mode. Data were low-pass–filtered at 30 kHz (−3 dB, 4-pole Bessel filter) and digitized at 100 kHz using a Digidata 1322A digitizer driven by PClamp 9 software (Molecular Devices, CA). After establishing whole cell configuration, neurons were dialyzed for 20 min to 1 h. Recordings were performed at either room temperature or at 32 °C.

### 2.2 High-speed fluorescence imaging using laser spot illumination

SBFI fluorescence was excited using a digitally modulated, ultra-low-noise laser (377 nm, Stradus Versalase multi-wavelength laser system, Vortran Laser Technology, CA), controlled via Stradus software. The same laser was used for bis-fura-2. ANG-2 was excited with a 517 nm laser. OGB-1 or OGB-5 N fluorescence was excite by a 476 nm laser (Miyazaki and Ross, 2017, 2022). The beam was delivered through a modified AiWon device (Rapp OptoElectronic, Wedel, Germany) and focused onto the tissue, creating an illumination spot of ~20–100 μm, depending on the fiber optic diameter and the lens. The emission light was collected using a custom Chroma C180410 filter or a custom-made double band filter (Miyazaki and Ross, 2017, 2022). Changes in fluorescence were acquired at 500–1000 frames/s using a back-illuminated 80 × 80 pixel cooled camera (NeuroCCD-SMQ; RedShirt Imaging, GA) controlled by Neuroplex software (RedShirt Imaging, GA). Quasi-simultaneous optical recording of SBFI and OGB-1 transients were obtained by delivering trains of alternating 200–400 μs long laser pulses aligned with the 1 ms frames of the CCD camera (Miyazaki and Ross, 2017).

### 2.3 Modeling

Numerical simulations were performed in the NEURON environment (Hines and Carnevale, 1997, 2000). Unless otherwise stated, electrophysiological parameters and dynamic [Na^+^]_i_ changes were studied in a simplified compartmental model that encompassed the fundamental morphological and electrical features of Layer 5 pyramidal neurons, as described previously (Fleidervish et al., 2010; Baranauskas et al., 2013). In the model, the 1 μm-thick axon initial segment (AIS) extended over the first 50 μm of the axon. The subsequent segment (length, 50 μm; diameter, 1 μm) was myelinated and it was separated from the first node of Ranvier (length, 1 μm) by a paranode (length 1 μm, resistivity 5∙10^7^ M*Ω*/cm). In some models the node had longer lengths (up to 10 μm). The next eight myelinated internodes were 25 μm long. The soma (length, 23 μm; diameter, 23 μm) gave rise to a single apical dendrite (length, 700 μm; diameter, 3.5 μm) and to two basal dendrites (length, 200 μm; diameter, 1.2 μm). The passive electrical properties *R*_m_, *C*_m_ and *R*_i_ were set to 15,000 Ω cm^2^, 0.9 μF cm^−2^ and 150 Ω cm, respectively, uniformly throughout all compartments. Myelination was simulated by using Neuron extracellular mechanism (Hines and Carnevale, 1997, 2000). The kinetics and distribution of Na^+^ channels were modeled as previously described (Fleidervish et al., 2010; Baranauskas et al., 2013). High-threshold Ca^2+^ channels with generic kinetics (Destexhe et al., 1993) were incorporated exclusively at the nodes. The resting membrane potential at the soma was set to −75 mV. All simulations were run with 5-μs time steps and the nominal temperature of simulations was 32 °C.

The diffusion of Na^+^ ions was modeled as ion exchange between adjacent neuronal compartments using the protocols in NEURON, assuming a diffusion coefficient of 0.6 μm^2^ ms^−1^ (Kushmerick and Podolsky, 1969; Fleidervish et al., 2010). The resting intracellular and extracellular Na^+^ concentrations were set to 4 and 151 mmoL/L, respectively. Ca^2+^ buffering, radial and longitudinal diffusion, and active extrusion were modeled using a published NEURON mechanism (Holmes et al., 2017), with two modifications: the calcium diffusion coefficient was set to 0.014 μm^2^ ms^−1^ (Kushmerick and Podolsky, 1969), and the pump density to 10^−13^ mol/cm^2^, in order to match Ca^2+^ imaging results obtained with the OGB-5 N indicator. The resting Ca^2+^ concentration was set to 0.05 μM. For compartments receiving Ca^2+^ influx, the internal volume was divided into four concentric annuli.

## 3 Results

**Figure 1:**
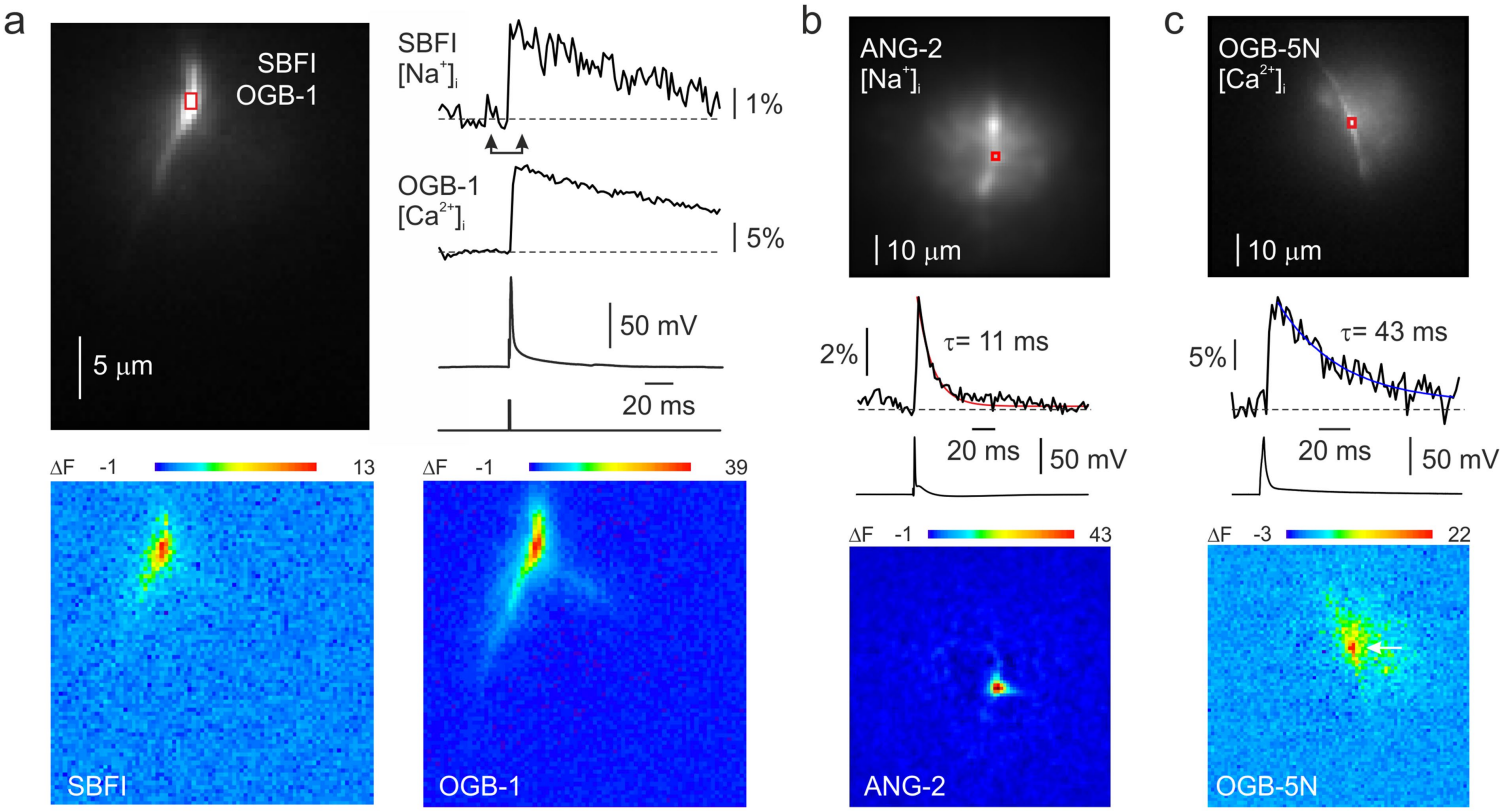
[Ca^2+^]_i_ and [Na^+^]_i_ changes in a node of Ranvier of L5 pyramidal neuron in response to somatically elicited action potential. **(a)** Quasi-simultaneous imaging of Na^+^ and Ca^2+^ transients using SBFI and OGB-1 indicators. Top, left: Fluorescence image of a small region of an axon of a L5 pyramidal neuron. Top, right: The changes in fluorescence related to changes in [Ca^2+^]_i_ and [Na^+^]_i_ in response to a single AP. Bottom: Difference images for SBFI and OGB-1 fluorescence change (ΔF) measured between the times of the arrowheads. The sodium trace and image are inverted because fluorescence of SBFI decreases when [Na^+^]ᵢ increases. **(b)** Imaging nodal [Na^+^]ᵢ dynamics using the Na^+^ indicator ANG-2 revealed a fast decay of the nodal Na^+^ signal. **(c)** Imaging nodal [Ca^2+^]_i_ dynamics using the low-affinity calcium indicator OGB-5 N.

We recorded fluorescence changes of Na^+^-sensitive and Ca^2+^-sensitive dyes evoked in the nodes of Ranvier of L5 pyramidal neurons by a single action potential. All the fluorescence transients were blocked by bath-applied tetrodotoxin (1 μM, *n* = 3). Nodes were identified by the site of localized Na^+^ transients (Fleidervish et al., 2010) and sometimes additionally by the presence of thin collateral axonal branches that originated from the axonal trunk at the same location. Figure 1a shows a representative quasi-simultaneous optical recording of the nodal SBFI and OGB-1 transients. Na^+^ transients, measured with the 2 ms frame rate of the camera, reached their peak at 3.7 ± 0.3 ms (*n* = 6) after the peak of the action potential, while Ca^2+^ transients exhibited a further delay, peaking at 10.3 ± 0.6 ms (*n* = 6). The spatial extent of the Na^+^ signals varied considerably between individual nodes, likely reflecting the broad range of nodal lengths (0.4–3.7 μm) reported in Layer 5 cortical myelinated axons immunolabeled for Na^+^ channels and Caspr (Arancibia-Cárcamo et al., 2017). As shown in the pseudo-color map of ΔF changes (Figure 1a, bottom), the spatial profiles of [Na^+^]_i_ elevation typically extended beyond the nodal region, consistent with lateral diffusion of Na^+^ ions into the adjacent internodal segments, often reaching 10–20 μm from the node. Ca^2+^ signals, which are expected to be more confined to the sites of Ca^2+^ influx because Ca^2+^ is buffered ~100x more strongly than Na^+^ (Kushmerick and Podolsky, 1969), were often broader than the Na^+^ signals in the same individual node, raising the possibility that Ca^2+^ channels may not be strictly confined to the nodal region. In several experiments, we monitored nodal [Na^+^]ᵢ dynamics using the green Na^+^ indicator Asante Natrium Green-2 (ANG-2) (Figure 1b). Similar to some experiments using SBFI, the ANG-2 transients were tightly confined to the presumed nodal region and exhibited rapid decay, likely reflecting shorter nodal lengths rather than differences in Na^+^ buffering capacity between ANG-2 and SBFI; both indicators have comparable Na^+^ binding affinities (Minta and Tsien, 1989; Roder and Hille, 2014). Since high-affinity Ca^2+^ indicators like OGB-1 can distort the temporal and spatial dynamics of Ca^2+^ signals, we recorded nodal Ca^2+^ transients using a low-affinity indicator (K_d_ ~ 20 μM), OGB-5 N (Figure 1c), which is expected to track the [Ca^2+^]_i_ transients more accurately. OGB-5 N transients reached their peak more rapidly (4.2 ± 0.5 ms, n = 6) and decayed significantly faster than those recorded with OGB-1 (decay time constant: OGB-1, 106 ± 8 ms, *n* = 7; OGB-5 N, 53 ± 4 ms, *n* = 6). Notably, while the decay kinetics of OGB-5 N transients were significantly faster, their spatial spread remained broader than that of the Na^+^ transients.

**Figure 2:**
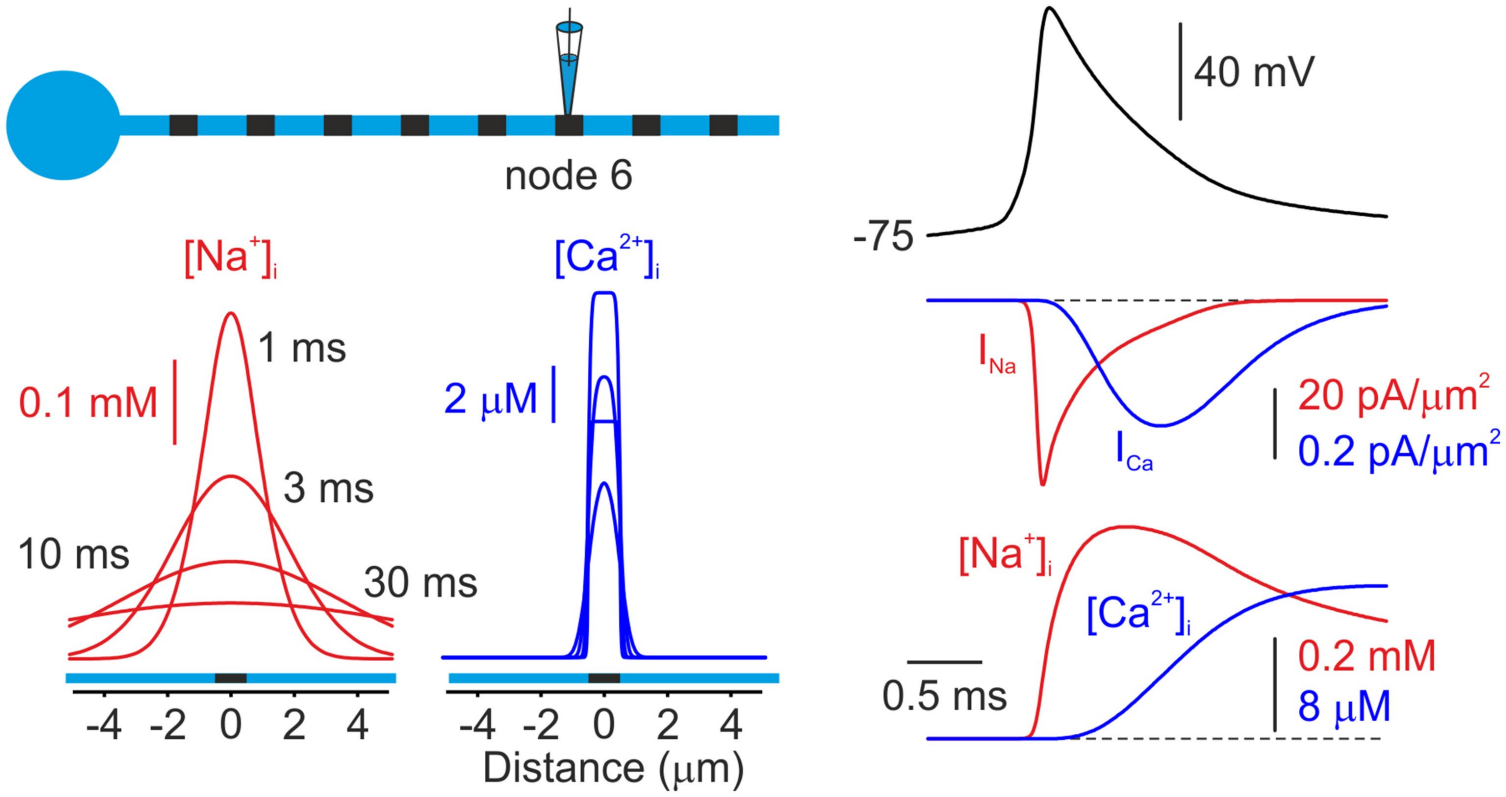
Model of Na^+^ and Ca^2+^ dynamics in a node of Ranvier. *Left*, Simulated changes in intracellular [Na^+^]ᵢ (red) and [Ca^2+^]ᵢ (blue) concentrations elicited by an action potential, plotted as a function of distance from the sixth node of Ranvier. Both concentrations are shown at times of 1,3, and 30 ms after the initiation of the AP. The node was modeled as 1 μm in length, with Na^+^ and Ca^2+^ channels localized to the nodal membrane. *Right*, Simulated currents and concentrations at the node following the initiation of an AP. Na^+^ channels activate primarily during the action potential upstroke and inactivate rapidly. Ca^2+^ currents exhibit delayed activation, reflecting the slower activation-deactivation kinetics of Ca^2+^ channels (“tail current”). The increases in concentrations at the node closely follow the integrals of the currents for the short times of this figure. Note that the peak of the [Na^+^]_i_ change occurs before I_Na_ returns to zero.

We next compared the action potential-associated nodal [Na^+^]_i_ and [Ca^2+^]_i_ changes with the dynamics calculated in a compartmental model that incorporates the basic morphological and physiological features of cortical myelinated axons (Figure 2). In the model, an action potential, elicited by a brief current pulse delivered to the soma, propagates in a saltatory manner through the sequence of myelinated internodes and nodes (Gow and Devaux, 2008; Cohen et al., 2020) driven by the activation of nodal Na^+^ channels. The Na^+^ current at the node activated rapidly during the action potential upstroke and fully inactivated during repolarization. In contrast, the Ca^2+^ current exhibited delayed activation, peaking at the end of repolarization, followed by rapid deactivation, consistent with voltage-clamp measurements (Augustine et al., 2003). We previously demonstrated that, in the nodes of Ranvier, as in other neuronal compartments, short-term [Na^+^]ᵢ dynamics are primarily governed by Na^+^ influx and lateral diffusion (Fleidervish et al., 2010). Notably, due to the exceptionally rapid diffusion of Na^+^ from the nodal volume into adjacent internodes, the model predicts that nodal [Na^+^]ᵢ is expected to reach its peak prior to the complete cessation of Na^+^ influx resulting from I_Na_ inactivation. The nodal [Ca^2+^]ᵢ, however, rises continuously during the Ca^2+^ influx and reaches its peak several milliseconds after the I_Ca_ peak, reflecting the combined effects of slow Ca^2+^ channel inactivation and pumping. Figure 2 (bottom) shows simulated changes in [Na^+^]ᵢ and [Ca^2+^]ᵢ elicited by a single action potential, plotted as a function of distance from the node at 1, 3, 10, and 30 ms following the spike peak. The model predicts that diffusion-mediated [Na^+^]ᵢ equilibration between the nodal and internodal volumes occurs within milliseconds, leading to a rapid decay of the nodal transient and a significant Na^+^ elevation in the adjacent internodes. Although the Ca^2+^ transients decay nearly as rapidly as the Na^+^ transients, they remain largely confined to the nodal volume, with minimal lateral dissipation, because they are controlled by pumps (Scheuss et al., 2006) instead of by diffusion.

**Figure 3:**
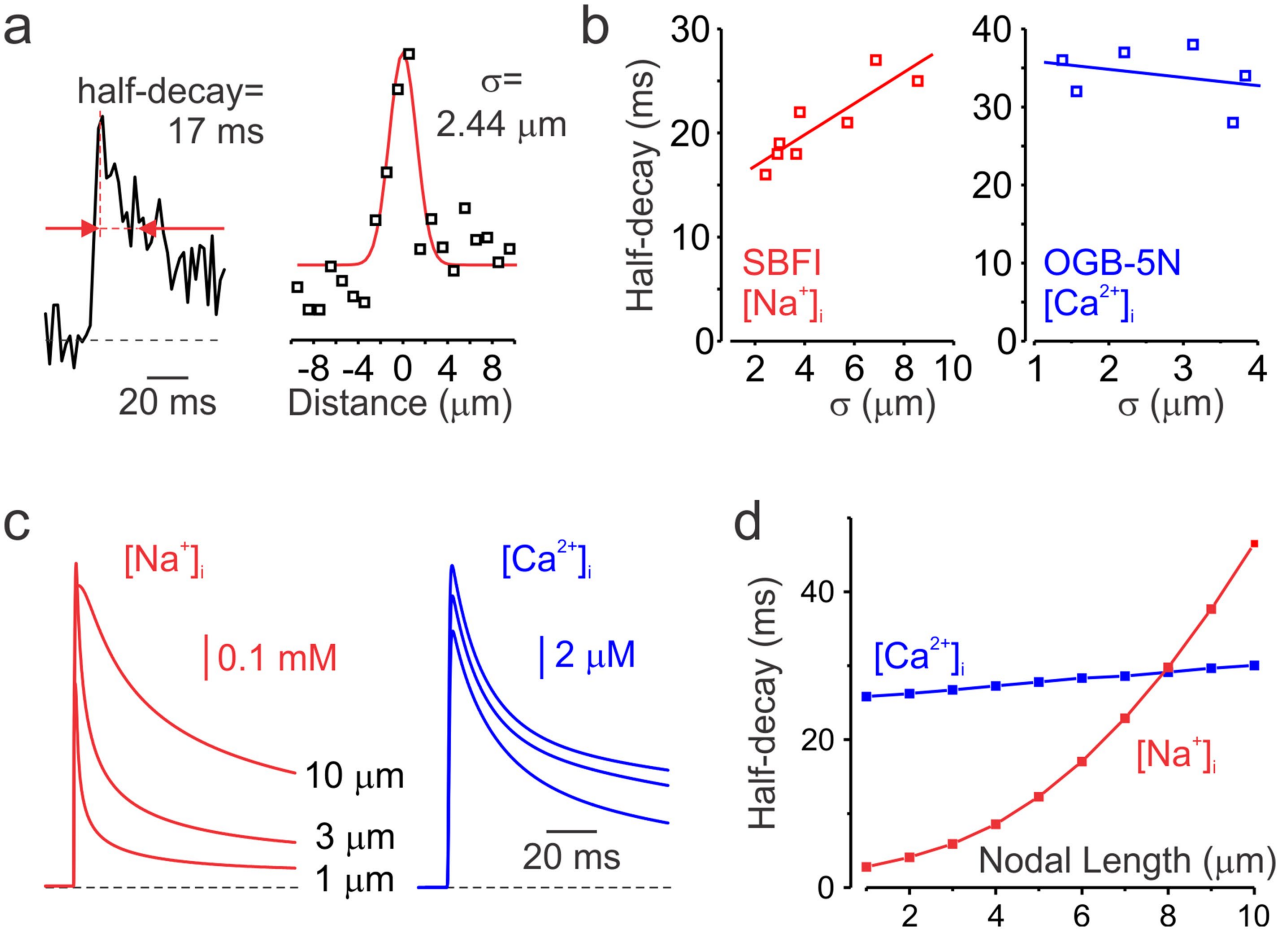
Decay kinetics of nodal Na^+^ and Ca^2+^ transients. **(a)** Measurements of the decay half-time and spatial spread of a representative nodal Na^+^ transient. *Left*, a representative averaged Na^+^ transient (*n* = 2) recorded at the center of the node of Ranvier. Red arrows indicate the time points corresponding to the transient’s half-amplitude width. *Right*, spatial extent of the same Na^+^ transient. Black squares represent the mean ΔF measured between 2 and 12 ms after the action potential peak. The red line shows a Gaussian fit to the ΔF–distance plot. *σ* = FWHM. **(b)** Half-decay times of Na^+^ (*n* = 8, red) and Ca^2+^ (*n* = 6, blue) transients plotted against their corresponding spatial extent. Continuous lines represent linear fits to the data. While Na^+^ transients decay more slowly in longer nodes, the time course of the decay of Ca^2+^ transients remains largely unchanged. **(c)** Simulated Na^+^ (red) and Ca^2+^ (blue) transients in models with nodal lengths of 1, 3, and 10 μm. Transients were sampled at 500 Hz to match the temporal resolution of the optical recordings. **(d)** Half-decay times of simulated Na^+^ (red) and Ca^2+^ (blue) transients plotted as a function of nodal length.

While the decay time course of nodal Ca^2+^ transients measured with the low-affinity Ca^2+^ indicator OGB-5 N was relatively consistent (half-decay time 34 ± 4 ms, mean ± SD; *n* = 6), the decay of Na^+^ transients measured with SBFI showed greater variability. Since the decay of Na^+^ transients reflects lateral diffusion of Na^+^ ions, which is influenced by the highly variable nodal length (Arancibia-Cárcamo et al., 2017), we tested the hypothesis that nodes exhibiting slower decay kinetics are longer than those with faster decay. As direct measurement of nodal length in live preparations is not feasible, we estimated it indirectly by quantifying the spatial width (*σ*) of the ΔF signal surrounding the node during the 2–12 ms window following the action potential peak (Figure 3a). Analysis of half-decay times from single-pixel transients recorded at the center of individual nodes, plotted against the spatial width of both Na^+^ and Ca^2+^ transients (Figure 3b), revealed that Na^+^ decay kinetics correlated with the presumed nodal length, whereas no such relationship was observed for Ca^2+^ signals. In good agreement with the experimental results, simulations using computational models with varying nodal lengths predicted that Na^+^ transients decay more slowly as nodal length increases, whereas the decay of Ca^2+^ transients remains relatively constant (Figures 3c,d).

**Figure 4:**
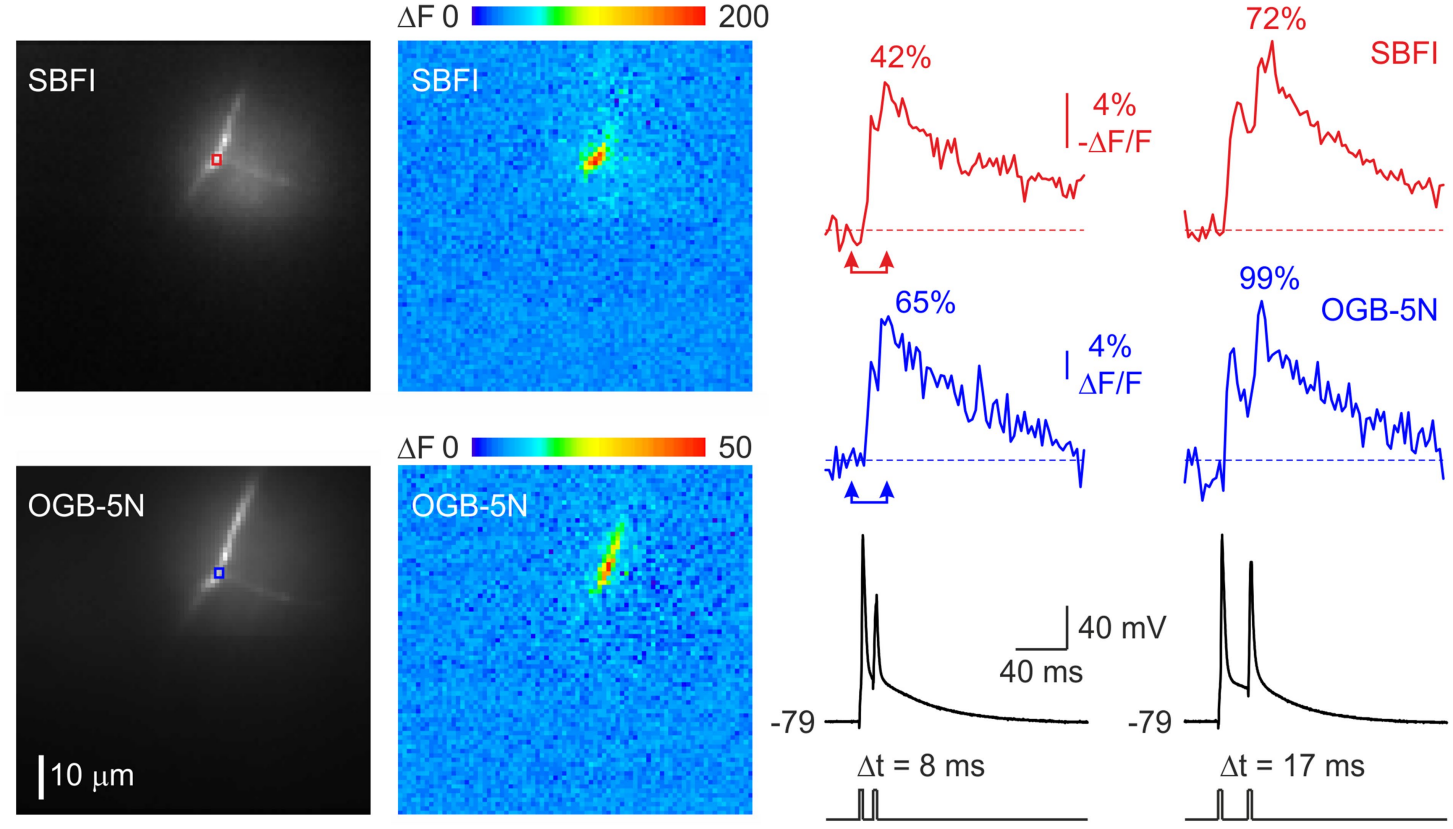
Rapid recovery of Na^+^ and Ca^2+^ fluxes at the node of Ranvier. Changes in [Na^+^]ᵢ (red) and [Ca^2+^]ᵢ (blue) concentrations elicited by a train of two action potentials delivered at intervals of 8 ms and 17 ms. The pseudocolor images show the spatial distribution of the distribution of the fluorescence changes between the times of the two arrowheads. It is noteworthy that in this experiment, during repetitive spike firing, Ca^2+^ flux, measured as the spike evoked amplitude of the fluorescence change, recovered more rapidly than Na^+^ flux. As noted in Figure 1d, this reversal can occur with longer nodes.

Faithful propagation of high-frequency spike trains in cortical myelinated fibers requires rapid recovery of nodal Na^+^ channels from inactivation. Standard voltage-clamp protocols used to evaluate channel kinetics are, however, not easy to apply to nodal Na^+^ channels due to the difficulty of controlling nodal membrane voltage from a remote somatic recording pipette. Therefore, we focused on comparing the amplitudes of nodal [Na^+^]_i_ changes evoked by pairs of action potentials delivered at varying interspike intervals, reasoning that the response to the second spike would be governed by the inactivation induced by the first spike. The fluorescence change is expected to be linear with Na^+^ influx if the measurement is made quickly before Na^+^ diffuses far from the node, since SBFI is a low affinity indicator. In a representative node (Figure 4), the [Na^+^] change elicited by the second action potential recovered to 42% at a spike interval of 8 ms, and to 72% at a 17 ms interval. The actual channel recovery rate may be even faster, as the Na^+^ influx during the second action potential should be reduced due to a smaller spike amplitude, assuming the amplitudes at the node correspond to the amplitudes measured by the somatic pipette. Recovery of spike-evoked [Ca^2+^]_i_ was even faster than that of [Na^+^]_i_, reaching 65% at 8 ms and 99% at 17 ms. Since Ca^2+^ channels are not expected to inactivate during a brief action potential, the reduced amplitude of the transient elicited by the second action potential most likely reflects a decrease in spike amplitude at the node, resulting from incomplete recovery of Na^+^ channels.

**Figure 5:**
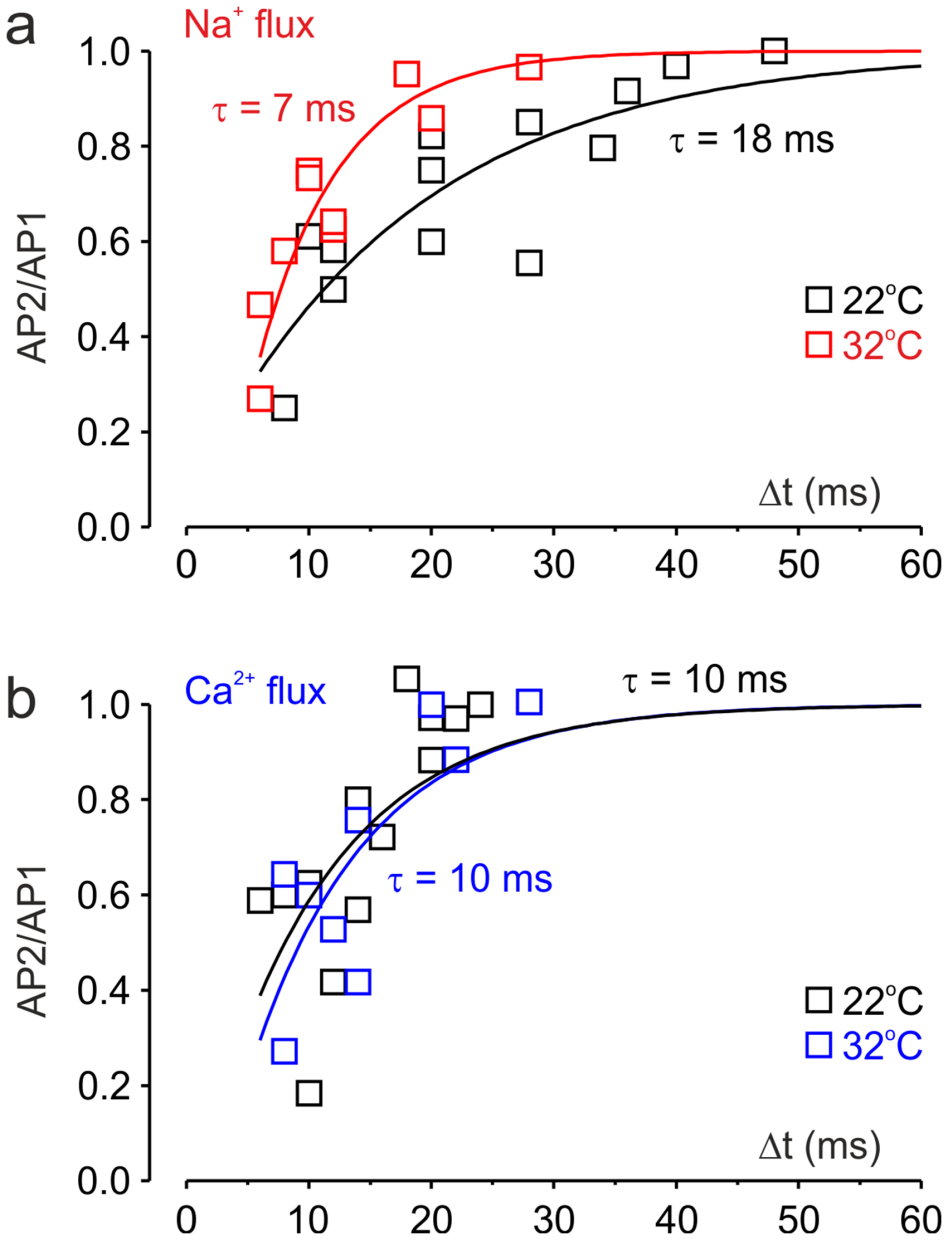
Effect of temperature on the recovery of nodal Na^+^ and Ca^2+^ fluxes. **(a)** Exponential fits of the ratio of peak Na^+^ transients elicited by the first and second action potentials at varying intervals. Na^+^ flux recovered with time constants of *τ* = 7 ms at 32 °C (red) and τ = 18 ms at 22 °C (black). The data are pooled recordings from five neurons. **(b)** Exponential fits of the ratio of peak Ca^2+^ transients. Nodal Ca^2+^ flux recovered with a time constant of τ = 10 ms at both 32 °C (blue) and 22 °C (black).

Figures 5a,b show the recovery kinetics of [Na^+^]_i_ and [Ca^2+^]_i_ levels, at temperatures of 22 °C and 32 °C. Squares represent signal ratios measured in five individual nodes, while the continuous lines show single-exponential fits to the pooled data. The effective recovery time constant of [Na^+^] was 7 ms at 32 °C and 18 ms at 22 °C, consistent with a Q_10_ of approximately 3, as previously reported for the inactivation kinetics of Na^+^ conductance at the squid giant axon (Hodgkin and Huxley, 1952) and somatic Na^+^ channels in cortical pyramidal neurons (Astman et al., 2006). In contrast, the recovery of [Ca^2+^] showed no apparent temperature dependence and was characterized by a time constant of 10 ms.

## 4 Discussion

This study utilized fluorescence imaging to examine the dynamics of Na^+^ and Ca^2+^ concentration changes at proximal nodes of Ranvier of L5 pyramidal neurons, revealing key differences in their temporal and spatial profiles. First, we found that the time-to-peak of nodal Na^+^ transients was significantly shorter than that of Ca^2+^ transients, consistent with electrophysiological evidence that Na^+^ influx occurs primarily during the action potential upstroke (Bean, 2007), whereas Ca^2+^ influx takes place mainly during and after the repolarization phase (Augustine et al., 2003; Ma et al., 2023). We also identified divergent mechanisms governing their clearance from the node. The decay of [Na^+^] from the node is primarily by diffusion of Na^+^ away from the node. Consequently, a longer node, with Na^+^ entry over a more extensive region, slows this process, which explains the positive correlation between nodal length and [Na^+^] decay time. The decay of the Ca^2+^ signal, however, is more complex, reflecting the combined effects of buffering, diffusion, and active pumping. The lack of correlation between nodal length and Ca^2+^ decay time aligns with prior evidence that Ca^2+^ diffusion in the axoplasm is markedly slower than that of Na^+^ (Kushmerick and Podolsky, 1969).

Our evidence indicates that the post-spike recovery of nodal Na^+^ fluxes was rapid and temperature-dependent, in agreement with the reactivation kinetics of Na^+^ channels (Fleidervish et al., 1996). The similarly rapid but temperature-independent recovery of Ca^2+^ flux supports the idea that a single action potential does not induce inactivation of Ca^2+^ channels (Borst and Sakmann, 1998) and thus has minimal impact on their availability for subsequent spikes. The transient reduction in the amplitude of nodal Ca^2+^ transients at short inter-spike intervals is most likely due to decreased Ca^2+^ influx during the smaller second spike. This, in turn, reflects incomplete recovery of Na^+^ channels, leading to reduced excitability and a diminished amplitude of the second AP. Importantly, this secondary reduction in Ca^2+^ entry does not imply direct modulation of Ca^2+^ channels, but rather underscores the critical dependence of Ca^2+^ influx on the integrity of the spike waveform.

The kinetics of nodal ion channel currents are subject to debate regarding their resemblance to other neuronal compartments. Our findings, however, provide strong evidence that the kinetics are similar to kinetics measured at the soma (Fleidervish et al., 1996; Augustine et al., 2003; Bean, 2007; Ma et al., 2023). For example, presynaptic Ca^2+^ channels exhibit slower activation and inactivation kinetics than Na^+^ channels, leading to prominent tail currents like those observed at the node (Augustine et al., 2003). Furthermore, the recovery time constant for Na^+^ flux at the node aligns with previous reports on the inactivation kinetics of somatic Na^+^ channels (Fleidervish et al., 1996).

Immunohistochemical labeling (Arancibia-Cárcamo et al., 2017; Eshed-Eisenbach and Peles, 2021; Rasband and Peles, 2021), along with our previous functional Na^+^ imaging study (Fleidervish et al., 2010), consistently demonstrate that Na^+^ channels, predominantly of the Nav1.6 subtype (Boiko et al., 2001), are confined to the nodal membrane and are not present in the internodal axolemma. A recent voltage imaging study (Cohen et al., 2020) demonstrated that during an action potential, the internodal axolemma undergoes a substantial depolarization of approximately 50 mV, sufficient to drive significant Na^+^ influx if Na^+^ channels were present beneath the myelin. Furthermore, the observed amplitude, spatial distribution, and temporal dynamics of internodal [Na^+^] elevations were well accounted for by a diffusional model and did not necessitate local Na^+^ influx. While Ca^2+^ channels are known to be present along unmyelinated axons (Westenbroek et al., 1995), their precise localization and distribution within myelinated axons are not well understood. Fluorescent Ca^2+^ imaging reports uniform Ca^2+^ elevations in myelinated axons of mouse optic nerve (Zhang et al., 2006), whereas Ca^2+^ transients confined to the presumed nodes of Ranvier were reported in axons of mouse cerebellar Purkinje neurons (Grundemann and Clark, 2015) and L5 pyramidal axons (Hanemaaijer et al., 2020). Although Ca^2+^ elevations typically exhibited a broader spatial extent than Na^+^ signals, this difference may reflect the lateral diffusion of Ca^2+^ ions and the higher sensitivity of Ca^2+^ indicators. Our findings are broadly consistent with the co-localization of Na^+^ and Ca^2+^ channels at the node of Ranvier; however, the presence of functional Ca^2+^ channels in the paranodal and proximal internodal regions cannot be excluded.

AP-associated Ca^2+^ influx in the AIS has been shown to be partially mediated by Nav1.2 channels, which exhibit a measurable permeability to Ca^2+^ ions (Hanemaaijer et al., 2020; Filipis et al., 2023). The temporal resolution of our nodal recordings, however, does not allow to resolve any potential contribution of Na^+^ channels to the Ca^2+^ transients. Although the nodes of Ranvier in mature rodent axons are predominantly populated by Ca^2+^-impermeable Nav1.6 channels (Boiko et al., 2001; Kaplan et al., 2001), a contribution of Na^+^ channels to nodal Ca^2+^ influx cannot be ruled out.

A primary limitation of this study was the inherent trade-off between temporal and spatial resolution. At a frame rate of 500 Hz, the CCD camera effectively integrated the fluorescence signal over 2 ms intervals, resulting in temporal binning of the data. This binning, coupled with the diffusion of Na^+^ ions following influx, likely resulted in a distorted overestimation of the sodium channel spatial extent and, consequently, the node length. To address these limitations, we developed a computational model incorporating binned sodium influx data for varying nodal lengths, allowing a more accurate estimation of the nodal length. However, this corrected model still does not account for the observation that Na^+^ entry often extends for more than 1 μm. Rather, our results strongly suggest that many nodes, especially those close to the soma, are longer. Nodes longer than 1 μm have been observed in other axons (Arancibia-Cárcamo et al., 2017).

## Data Availability

The raw data supporting the conclusions of this article will be made available by the authors, without undue reservation.
